# Renal MRI radiomics in Beckwith–Wiedemann syndrome: a novel imaging approach for genotype identification

**DOI:** 10.1186/s13023-025-03841-x

**Published:** 2025-06-15

**Authors:** Mei Bai, Xiansheng Wu, Jinghui Wang, Miaoying Zhang, Zhongwei Qiao, Lin Zhang, Jungang Liu

**Affiliations:** 1 Department of Radiology, Children’s Hospital of Fudan University, Shanghai, China; 2 Department of Radiology, Xiamen Children’s Hospital (Children’s Hospital of Fudan University Xiamen Branch), No 92-98, Yibing Road, Huli District, Xiamen, Fujian China; 3 Department of Clinical Laboratory, Xiamen Children’s Hospital (Children’s Hospital of Fudan University Xiamen Branch), No 92-98, Yibing Road, Huli District, Xiamen, Fujian China; 4 Department of Endocrinology, Children’s Hospital of Fudan University, Shanghai, China

**Keywords:** Radiomics, Beckwith–Wiedeman syndrome, Kidney, Genotype

## Abstract

**Purpose:**

To valuate the role of nonmalignant nephrological findings and renal MRI radiomics in differentiating molecular subtypes of Beckwith–Wiedemann syndrome (BWS).

**Materials and methods:**

Clinical data and abdominal MRI scans of 49 patients who underwent partial glossectomy between July 2019 and March 2024 were retrospectively analysed. Patients were categorized into two subtypes: BWS^UPD+IC1^ (24 cases, with a predisposition to renal involvement) and BWS^IC2^ (25 cases, with a lower risk of renal involvement), based on genetic testing. Pearson correlation analysis was conducted to evaluate the relationship between patients’ age and renal volume. Radiomic features derived from the T2WI sequence and the ADC map were selected to construct single-sequence and combined models. Delong test was used to compare the performance of the models.

**Results:**

Clinically, the BWS^UPD+IC1^ subtype exhibited a lower incidence of ear creases/pits (*P* = 0.048) and omphalocele/umbilical hernia (*P* = 0.032) compared to the BWS^IC2^ subtype. Abdominal MRI findings indicated the BWS^UPD+IC1^ subtype had larger total renal volume (*P* = 0.017) and a weaker correlation between total renal volume and patients’ age (r = 0.38). Notably, 91.84% (45/49) of BWS patients exhibited a total renal volume exceeding the normal population’s upper limit, with the IC1 subtype demonstrating the largest mean volume. The BWS^UPD+IC1^ subtype showed higher incidences of nonmalignant renal (*P* = 0.013) and non-renal abdominal abnormalities. The T2WI, ADC, and combined models achieved the highest area under the receiver operating characteristic (ROC) curves (AUCs) of 0.837, 0.882 and 0.954 (*P* > 0.05), respectively.

**Conclusion:**

Nonmalignant renal abnormalities and MRI radiomics models have potential as alternative imaging tools for the identification of renal predisposition genotypes and the surveillance of renal size change in BWS patients.

**Supplementary Information:**

The online version contains supplementary material available at 10.1186/s13023-025-03841-x.

## Introduction

Beckwith–Wiedemann syndrome (BWS) is an overgrowth and multisystem human genomic imprinting disorder characterized by variable clinical phenotypes and a complex molecular etiology [1]. The main clinical features of BWS include pre- and postnatal constitutional and organ overgrowth, macroglossia, omphalocele/umbilical hernia, facial nevus flammeus, hemihyperplasia, and embryonal tumors [2]. The molecular genotypes of BWS encompass loss of imprinting at the region of imprinting control 2 (IC2) in 50% of cases, paternal uniparental disomy (UPD) of chromosome 11p15 in 20%, gain of imprinting at IC1 in 5–10%, and mutations of the maternal CDKN1C gene in about 5% of sporadic cases and 40% of familial cases [3]. Many previous studies have delineated distinct clinical phenotypes associated with BWS molecular subtypes across diverse populations [4–7], which could serve as valuable indicators for differentiating molecular subtypes. Zhang et al. reported that the existing clinical markers for BWS had a 70% positive predictive value for a positive epigenetic test outcome [8].

Kidney-related anomalies are a significant clinical feature in BWS, with a notable incidence of 25% for renal nonmalignant lesions [9] and 4.1% for Wilms tumor [10]. Some studies have focused on the correlation between renal tumor risk and the genetic or epigenetic cause of BWS, revealing that the risks of Wilms tumor are notably higher for the IC1 and UPD subtypes, at 24% and 7.9% respectively, while the IC2 subtype presents a significantly lower risk of 0.2% [11]. However, few studies have explored the association between nonmalignant nephrological findings and molecular subtypes of BWS. By using abdominal ultrasound, one study has indicated that BWS^UPD^ is associated with a high incidence of benign lesions in the kidney compared to BWS^IC2^ [12]. Further research has identified the correlation between BWS^UPD+IC1^ and nephrological findings [13]. Additionally, nephromegaly as a nonmalignant nephrological finding has been correlated with the risk of Wilms tumor in BWS [12, 14], which highlights the pressing need to better understand the relationship between different genetic subtypes of BWS and specific renal nonmalignant manifestations. The advent of multiparametric MRI, such as the combination of anatomical imaging (e.g., T2-weighted imaging [T2WI]) and functional imaging (e.g., diffusion-weighted imaging [DWI]), along with radiomic analysis, has significantly contributed to the assessment of renal morphology, microstructure, and function [15, 16]. This advancement may potentially aid in distinguishing BWS genotypes.

In this study, we aimed to establish the correlation between BWS genotype and the renal image phenotype. A cohort of 49 BWS patients with three common genotypes was divided into two subtypes based on the renal predisposition for nonmalignant lesions: BWS^UPD+IC1^ (renal predisposition subtype) and BWS^IC2^ (renal non-predisposition subtype), as determined by multiplex ligation-dependent probe amplification (MLPA) testing. Renal MRI examinations, including T2WI and DWI sequences were performed, and renal radiomics were applied to analyse the renal features and construct models to differentiate the BWS genetic subtypes. This study could lead to more tailored surveillance strategies for BWS patients.

## Methods

### Patient selection

**Fig. 1:**
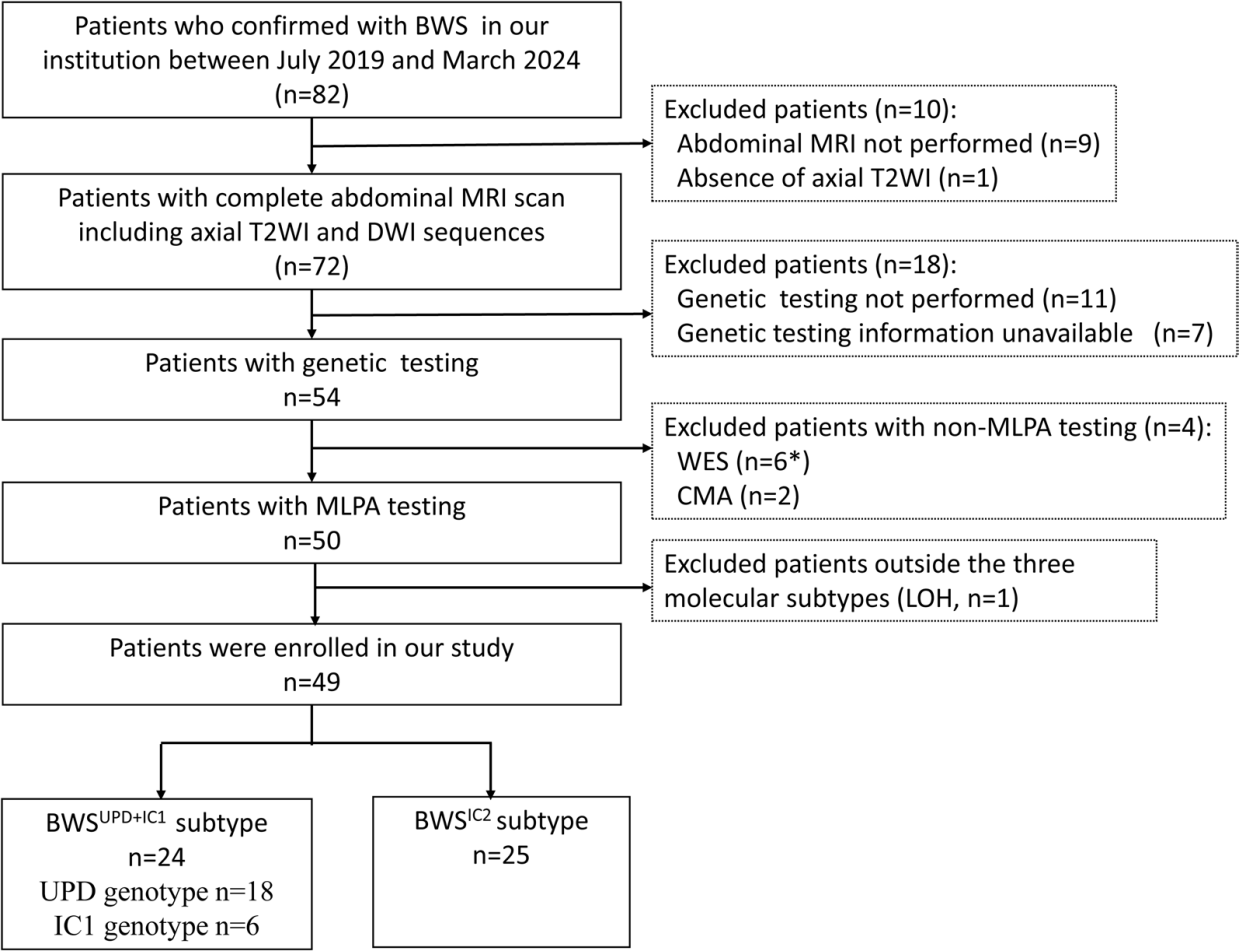
The flowchart of patient recruitment. *4 patients underwent both WES and MLPA testing, 1 patient was CDKN1C, 1 patient was negative result. WES, whole exome sequencing; CMA, chromosomal microarray analysis; MLPA, multiplex ligation-dependent probe amplification; LOH, loss of heterozygosity

This retrospective study was approved by the institutional research ethics committee. Informed consent was acquired from all patients included in this study. Between July 2019 and March 2024, 82 patients diagnosed with BWS were referred to our hospital for partial glossectomy due to macroglossia. Their renal MRI data and genetic testing were reviewed. Inclusion criteria were (1) completion of an abdominal MRI scan, including T2WI and DWI sequences, and (2) molecular subtype confirmation (IC1, IC2 and UPD) through MLPA testing. Excluded criteria included the absence of required MRI images (n = 10), lack of genetic testing (n = 18), use of non-MLPA methods for genetic analysis (n = 4), and genetic findings outside the three subtypes (n = 1). Finally, 49 patients were included, comprising 24 classified as BWS^UPD+IC1^ (6 IC1 cases and 18 UPD cases) and 25 classified as BWS^IC2^. The flow chart of our study is shown in Fig. 1.

### MRI acquisition

Abdominal MR imaging for all patients during the study period was performed with a 3.0 T MRI system (Magnetom Avanto; Siemens, Erlangen, Germany). To obtain anatomical and functional images of the kidneys, the imaging protocol for bilateral kidneys included axial turbo spin-echo (TSE) fat-suppressed T2WI and echo planar imaging (EPI) DWI with b-values of 0 and 800 mm^2^/s. An automatic apparent diffusion coefficient (ADC) map was generated for each examination. Given the young age of the participants, all imaging was performed under anaesthesia. The details of the applied parameters of MR imaging are presented in Table 1.

**Table 1:** Scan Parameters at 3.0 T MRI for BWS patients

Parameters	T2WI	DWI
Sequence name	TSE	EPI
Physiology (Breath technique)	Respiratory triggered	Breath free
Fat suppression	Yes	Yes
Repetition time(TR, msec)	2000	6600
Echo time (TE, msec)	78	54
Flip angle (degree)	87	–
Slice orientation	Axial	Axial
Slice thickness (mm)	4	4
Interslice gap (mm)	1	1
Echo train length	12	–
Field of view (FOV, mm^2^)	240*240	240*192
Acquisition matrix	256*256	100*100
Bandwidth (Hz/pixel)	814	2174
B-values (mm^2^/s)		0, 800

T2WI, T2-weighted imaging; DWI, diffusion-weighted imaging; TSE, turbo spin-echo; EPI, echo planar imaging

### Radiomics model construction

**Fig. 2:**
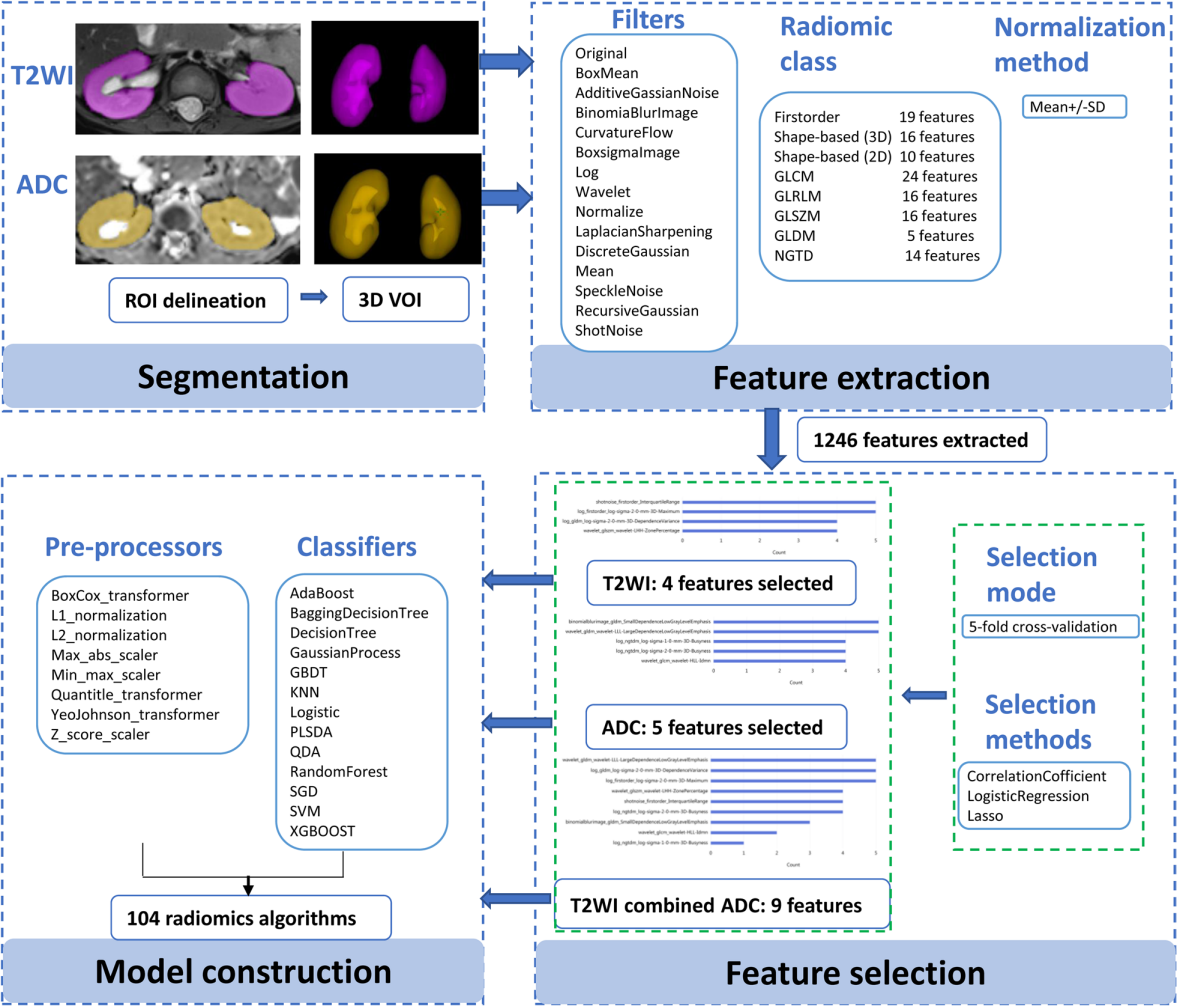
Radiomics workflow of this study. ROI, region of interest; VOI, voxel of interest; GLCM, gray level of co-occurrence matrix; GLRLM, gray level of run length matrix; GLSZM, gray level of size zone matrix; GLDM, gray level of dependence matrix; NGTDM, neighbouring gray tone difference matrix; GBDT, gradient boosting decision tree; KNN, k-nearest neighbors; PLSDA, partial least squares discriminant analysis; QDA, quadratic discriminant analysis; SGD, stochastic gradient descent; SVM, support vector machine

Radiomics workflow is presented in Fig. 2, including image segmentation, feature extraction, feature selection and model construction. All processes were performed using uAI software (United Imaging Healthcare, China). Regions of interest (ROIs) for the bilateral renal parenchyma were manually delineated slice by slice by a pediatric radiologist with 14-year experience in abdominal imaging (M.B), which then generates the voxels of interest (VOIs) based on T2WI and ADC maps. During the delineation, the ROIs excluded the renal collecting system, focusing exclusively on parenchymal features. Image segmentation was supervised by a senior pediatric radiologist with 23-year experience (XS.W). From the delineated ROIs, total renal volumes, mean T2WI signal intensities and ADC values were automatically calculated. The total renal volume was obtained by adding together the right and left kidney volume. To better understand renal size change in BWS patients, we utilized total renal volume data from Leung’s study on normal children and calculated the volume variation range with mean ± 2 standard deviation (SD) for children aged 6 to 48 months (in half-year intervals) for comparison with our data [17].

Feature extraction was applied to 15 filters, such as log, wavelet, short noise and binomial blur image. 1246 features were generated, divided into first-order features, shape-based features, and five main texture features: gray level of co-occurrence matrix (GLCM), gray level of run length matrix (GLRLM), gray level of size zone matrix (GLSZM), gray level of dependence matrix (GLDM), and neighboring gray tone difference matrix (NGTDM). Data normalization was achieved using the mean ± SD.

Feature selection involved correlation coefficient (*P *values of 0.05), logistic analysis (*P *values of 0.1 for univariate and 0.05 for multivariate analysis), and the least absolute shrinkage and selection operator (LASSO) with an alpha-value of 0.05. Five-fold cross-validation was applied for data partitioning. Finally, a total of 4 features were selected for the T2WI model and a total of 5 features were retained for the ADC model. The overall 9 features were used for the combined model with features from the T2WI sequence and the ADC map. Model construction employed eight pre-processors and 13 machine learning modeling methods, generating 104 algorithms for each imaging sequence and the combined model. Receiver operating characteristic (ROC) curves were applied to assess all three models’ performance and identify the optimal algorithm.

### Statistical analysis

Clinical characteristics and MRI findings between the BWS^UPD+IC1^ and BWS^IC2^ subtypes were compared using unpaired t-tests for normally distributed variables and Mann–Whitney U tests for non-normally distributed variables. Categorical variables were analysed with Chi-square or Fisher’s exact tests. Correlations between patients’ age and total renal volume were assessed using Pearson correlation coefficients and scatter plots. The diagnostic performance of the T2WI, ADC, and combined models was evaluated using the area under the ROC curve (AUC), sensitivity, and specificity metrics. Delong tests were conducted to compare the performance of the three models.

Statistical analyses were performed using SPSS 17.0 (SPSS Inc.). Figures were generated with Python v3.11 and associated libraries, including matplotlib v3.6.0, NumPy v1.23.3, pandas v1.5.0, and seaborn v0.12.0. Comparisons were considered statistically significant at *P* < 0.05.

## Results

### Clinical manifestations

The median age at the time of MRI scan was 15 months (IQR: 11–19.25 months; range: 6–48 months) in the BWS^UPD+IC1^ and 15 months (IQR: 12–18 months; range: 8–33 months) in the BWS^IC2^ subtypes (*P* = 0.865). Among the BWS^UPD+IC1^, 10 were male (10/24, 41.67%), compared to 17 male patients (17/25, 68%) in the BWS^IC2^ subtype (*P* = 0.064).

The incidence of ear creases and/or pits was lower in the BWS^UPD+IC1^ (4/24, 16.67%) subtype than that in the BWS^IC2^ (10/25, 40%) subtype (*P* = 0.048). Similarly, the prevalence of omphalocele or umbilical hernia was also lower in the BWS^UPD+IC1^ (9/24, 37.5%) subtype compared to the BWS^IC2^ (17/25, 68%) subtype (*P* = 0.032). There were no statistically significant differences between the two subtypes in the incidence of hemihypertrophy, macrosomia or facial nevus flammeus (*P* > 0.05). Beyond the primary and secondary clinical features of BWS, three patients exhibited unique clinical histories involving vascular malformations. One patient was confirmed with hepatic vascular malformation and treated with propranolol. Another patient had a small hemangioma under the eye. The third patient presented with a mass on the back associated with vascular malformation. The demographic characteristics and clinical manifestations of patients are displayed in Table 2.

**Table 2:** Clinical and renal MRI characteristics of BWS^UPD+IC1^ and BWS^IC2^ subtypes

Characteristics	BWS^UPD+IC1^ subtype	BWS^IC2^ subtype	*P* values
No	24	25	
Age, months, Median [IQR]	15 [11–19.25]	15 [12–18]	0.865
Sex, male (%)	10 (10/24, 41.67%)	17 (17/25, 68%)	0.064
*Clinical manifestations*			
Macroglossia	24 (24/24, 100%)	25 (25/25, 100%)	–
Hemihypertrophy	11 (11/24, 45.83%)	9 (9/25,36%)	0.484
Macrosomia	11 (11/24, 45.83%)	6 (6/25, 24%)	0.108
Facial nevus flammeus	3 (3/24, 12.5%)	9 (9/25,36%)	0.056
Ear creases/ pits	4 (4/24, 16.67%)	10 (10/25, 40%)	0.048
Omphalocele/umbilical hernia	9 (9/24, 37.5%)	17 (17/25, 68%)	0.032
The total renal volume on the T2WI sequence (cm^3^), Median [IQR]	116.18 [100.76–142.1]	91.46 [79.47–107.85]	0.017
The mean ADC values (10^−3^mm^2^/s), Mean ± SD	1340.13 ± 67.53	1343.81 ± 75.81	0.73
T2 values of kidneys, Median [IQR]	368.81 [327.26–396.51]	378.77 [353.23–421.17]	0.289
*Nonmalignant renal anomalies, No. of patients*	9# (9/24, 37.5%)	2 (2/25, 8%)	0.013
Nephromegaly	3 (2UPD + 1IC1)	1	
Hydronephrosis	3 (2UPD + 1IC1)	0	
Cortical cysts	2 (1UPD + 1IC1)	1	
Medullary cysts	1 (UPD)	0	
Abnormal signals in kidneys	1 (UPD)	0	
*Non-renal abdominal anomalies, No. of patients*	7 (7/24, 29.17%,)	0	–
Abnormal signals in liver	5 (5/24, 20.83%, 4UPD + 1IC1)	0	
Pancreatic hypertrophy	1 (1/24, 4.17%, UPD)	0	
Mesenteric cyst	1 (1/24, 4.17%, UPD)	0	

#One patient exhibited two renal abnormalities (nephromegaly combined with cortical cyst)

### MRI findings

**Fig. 3:**
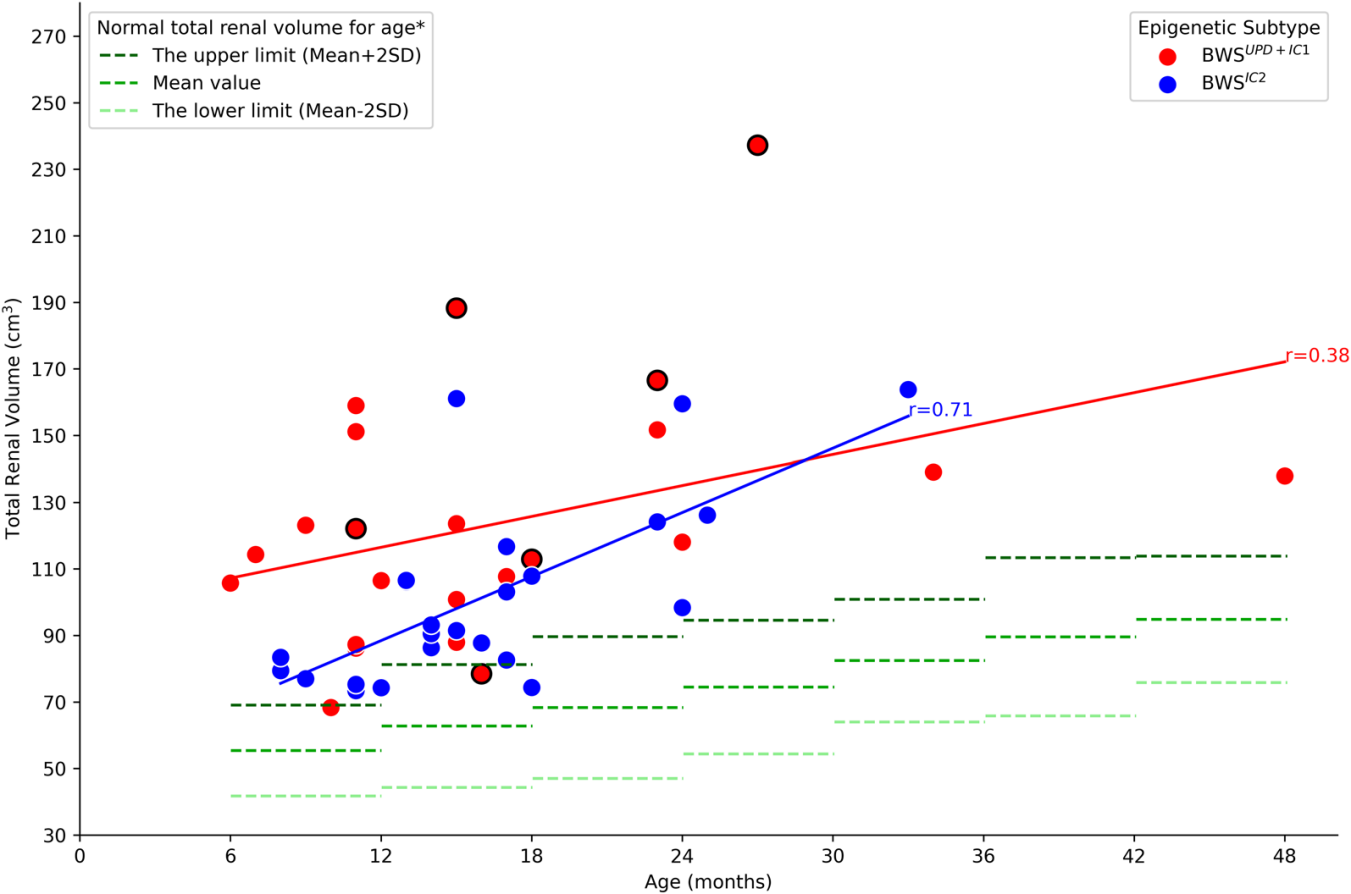
The variation range of total renal volume in normal children aged 6 to 48 months (in half-year intervals), along with the scatter plot and correlation between the total renal volume and the patient’s age in BWS patients. The three dashed lines, ranging from dark to light green, represented the upper limit (mean + 2SD), the mean value, and the lower limit (mean - 2SD) respectively. To distinguish between the UPD and IC1 subtypes, the red circular points for the IC1 subtype were outlined with black circles. Four patients had the total renal volume below the normal upper limit, which were one UPD patient (10 months), one IC1 patient (16 months), and two IC2 patients (12 and 18 months). *Based on reference [17]

On the T2WI sequence, the median total renal volume was significantly larger in the BWS^UPD+IC1^ (116.18 cm^3^) subtype than that in the BWS^IC2^ (91.46 cm^3^) subtype (*P* = 0.017). Furthermore, the correlation between the total renal volume and the patients’ age was significantly weaker in the BWS^UPD+IC1^ (r = 0.38) subtype than that in the BWS^IC2^ (r = 0.71) subtype. Compared to the normal population, 91.84% (45/49) of BWS patients, including 22 with BWS^UPD+IC1^ and 23 with BWS^IC2^, had a total renal volume exceeding the normal upper limit (dark green dashed line, mean + 2SD) in Fig. 3. In terms of mean total renal volume, there was a rough ranking of IC1 subtype > IC2 subtype > normal upper limit, while the UPD subtype, despite showing greater variation, still exceeded the normal upper limit (Table 3). There was no statistically significant difference between BWS^UPD+IC1^ and BWS^IC2^ in the ADC value (*P* = 0.73) and T2 intensity (*P* = 0.289).

**Table 3:** The total renal volumes in BWS patients and normal population

Age (months)	IC1 subtype		UPD subtype		IC2 subtype		Normal population*			
	No. of subjects	Mean value (cm^3^)	No. of subjects	Mean value (cm^3^)	No. of subjects	Mean value (cm^3^)	No. of subjects	The upper limit (Mean + 2SD) (cm^3^)	Mean value (cm^3^)	The lower limit (Mean-2SD) (cm^3^)
6–11.99	1	122.11	8	111.9	6	77.96	121	69.08	55.44	41.8
12–17.99	2	133.41	6	104.55	12	100	111	81.26	62.8	44.34
18–23.99	2	139.74	1	151.66	3	102.15	56	89.63	68.35	47.07
24–29.99	1	237.27	1	118.04	3	128.04	87	94.57	74.49	54.41
30–35.99	0	–	1	139.09	1	163.8	98	100.91	82.47	64.03
36–41.99	0	–	0	–	0	–	135	113.38	89.6	65.82
42–47.99	0	–	1	137.92	0	–	77	113.89	94.89	75.89

*Based on reference[17]

**Fig. 4:**
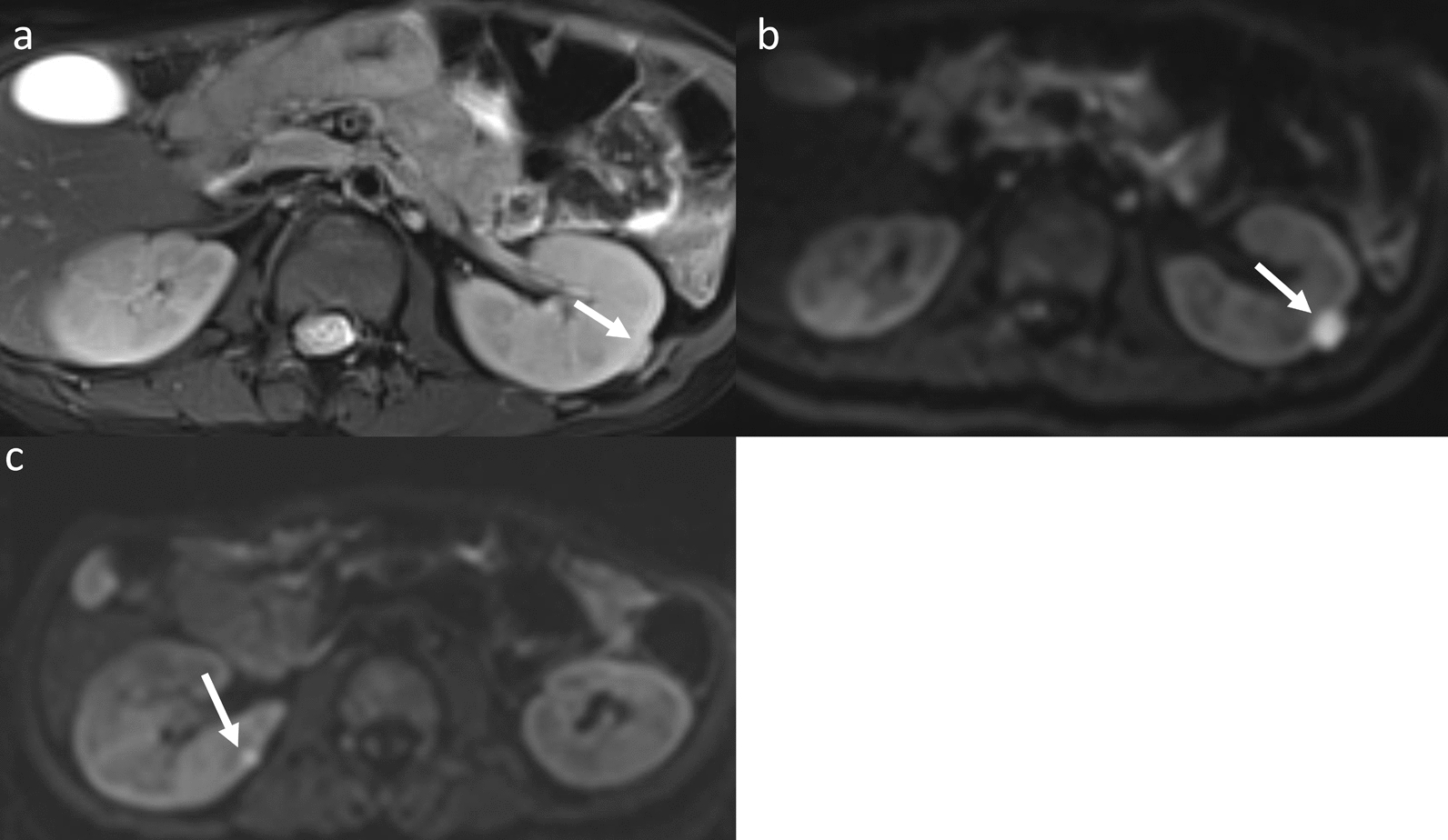
MRI of nephromegaly and complex cysts in a 2-year 3-month-old boy with BWS^IC1^ and a vascular malformation in the back. Arrows indicated an isointense signal on the fat-suppressed T2WI (a) and a hyperintense signal on the DWI (b) in the left kidney. A smaller hyperintense signal was observed in the right kidney on the DWI (c), which was not present on the T2WI

**Fig. 5:**
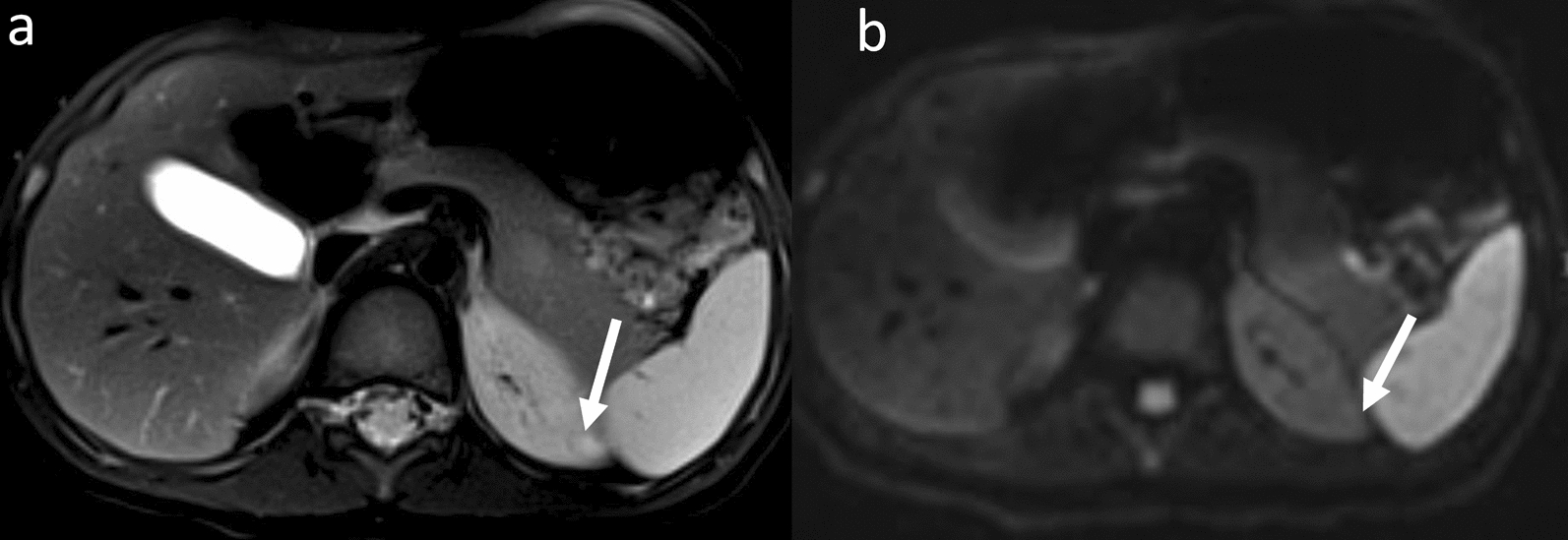
MRI of a simple cortical cyst in a 2 year, 9-month-old boy with BWS^IC2^. Arrows indicated a subtle hyperintense signal in the left kidney on the fat-suppressed T2WI (a). The lesion was invisible on DWI (b) and showed no diffusion restriction

Nonmalignant renal abnormalities occurred with an incidence of 22.45% (11/49 patients) among patients with the three main genetic errors (UPD, IC1 and IC2) in BWS, including nephromegaly (4/49, 8.16%), hydronephrosis (3/49, 6.12%), cortical/medullary cysts (4/49, 8.16%) and abnormal signals in kidneys (1/49, 2.04%; suspected as vascular malformations). Nonmalignant renal anomalies were more frequently observed in the BWS^UPD+IC1^ (9/24, 37.5%; 7 patients with UPD and 2 with IC1) subtype compared to the BWS^IC2^ (2/25, 8%) subtype (*P* = 0.013). In the BWS^UPD+IC1^, one patient with the IC1 subtype exhibited two renal abnormalities of nephromegaly and complicated cortical cysts in the kidneys and also had a vascular malformation in the back (Fig. 4). For the two IC2 subtype patients with nonmalignant renal abnormalities, one was nephromegaly, and the other was a subtle small simple cortical cyst in the left kidney which was less significant than those in the BWS^UPD+IC1^ subtype (Fig. 5).

**Fig. 6:**
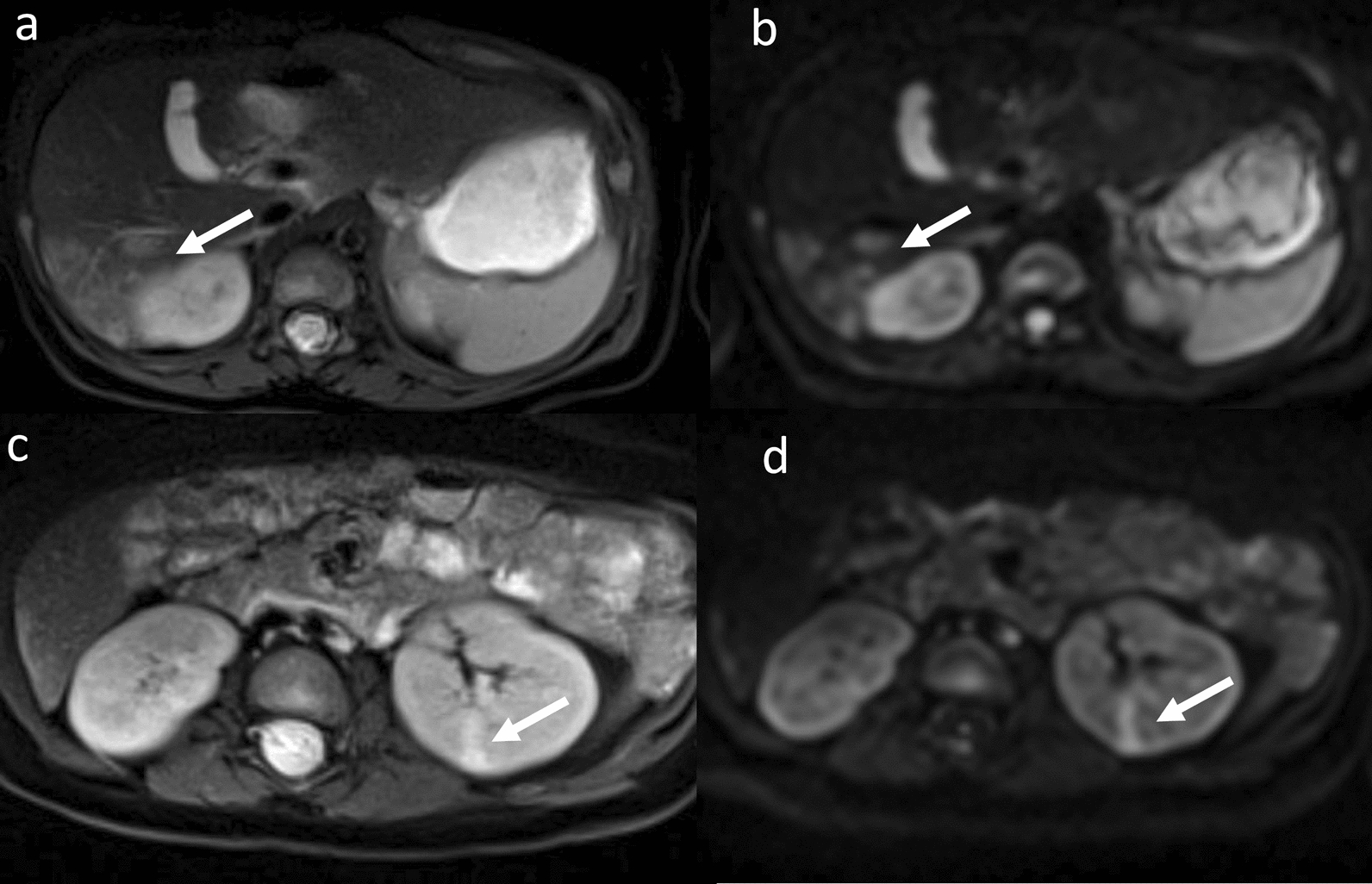
MRI of suspected multifocal vascular malformations in a 11-month-old girl with BWS^UPD^. Arrows indicated multiple patchy hyperintense signals in the liver on the fat-suppressed T2WI (a) and DWI (b). A similar signal was observed in the left kidney on T2WI and DWI (c, d)

**Fig. 7:**
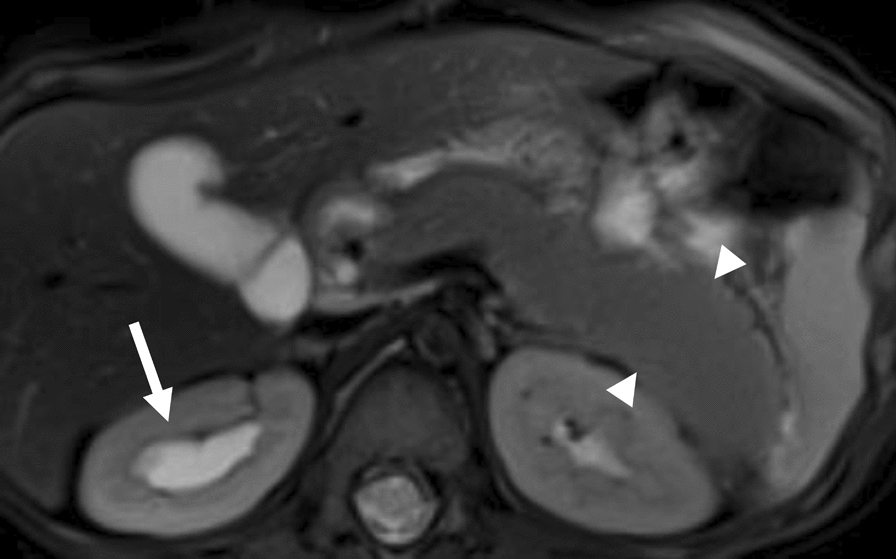
MRI of hydronephrosis and pancreatic hypertrophy in a 11-month-old boy with BWS^UPD^. The right renal hydronephrosis (arrow) and localized enlargement of the pancreatic body and tail (arrowhead) were observed on the fat-suppressed T2WI

7 patients (7/24, 29.17%) had non-renal abdominal abnormalities on MRI, which only occurred in the BWS^UPD+IC1^ subtype, including abnormal signals in the liver (5/24, 20.83%; 4 patients with UPD and 1 with IC1; suspected as vascular malformations), pancreatic hypertrophy (1/24, 4.17%) and mesenteric cyst (1/24, 4.17%). Among them, one patient with abnormal signals in both the kidneys and the liver (Fig. 6), was suspected of multifocal vascular malformation, one patient with pancreatic hypertrophy also had hydronephrosis (Fig. 7), and one patient with a hemangioma under the eye presented vascular malformation in the liver.

### Radiomics results

For the T2WI model, four selected features included two first-order features (with 5 counts) and two texture features (GLDM and GLSZM, with 4 counts). The ADC model consisted of five textural features: two GLDM features (with 5 counts), two NGTDM features (with 4 counts), and one GLCM feature (with 4 counts). In the combined model, which incorporated a total of 9 features, T2WI features had higher counts, including one first-order feature and GLDM (with 5 counts), as well as the other first-order feature and GLSZM (with 4 counts). In contrast, the ADC features had relatively variable counts, ranging from 1 to 5. The detailed information about selected features is shown in Table 4.

**Table 4:** Selected features for T2WI, ADC, and combined models

Selected radiomic features	MRI sequence	Voting count for the combined model	Voting count for a single model	Filters	Radiomics class
Wavelet_gldm_wavelet-LLL-LargeDependenceLowGrayLevelEmphasis	ADC	5	5	Wavelet	GLDM
Log_gldm_log-sigma-2–0-mm-3D-DependenceVariance	T2WI	5	4	Log	GLDM
Log_firstorder_log-sigma-2–0-mm-3D-Maximum	T2WI	5	5	Log	Firstorder
log_ngtdm_log-sigma-2–0-mm-3D-Busyness	ADC	4	4	Log	NGTDM
Wavelet_glszm_wavelet-LHH-ZonePercentage	T2WI	4	4	Wavelet	GLSZM
Shotnoise_firstorder_InterquartileRange	T2WI	4	5	Shot noise	Firstorder
Binomialblurimage_gldm_SmallDependenceLowGrayLevelEmphasis	ADC	3	5	Binomial blur image	GLDM
Wavelet_glcm_wavelet-HLL-Idmn	ADC	2	4	Wavelet	GLCM
log_ngtdm_log-sigma-1–0-mm-3D-Busyness	ADC	1	4	Log	NGTDM

GLDM, gray level of dependence matrix; NGTDM, neighbouring gray tone difference matrix; GLSZM, gray level of size zone matrix; GLCM, gray level of cooccurrence matrix

**Fig. 8:**
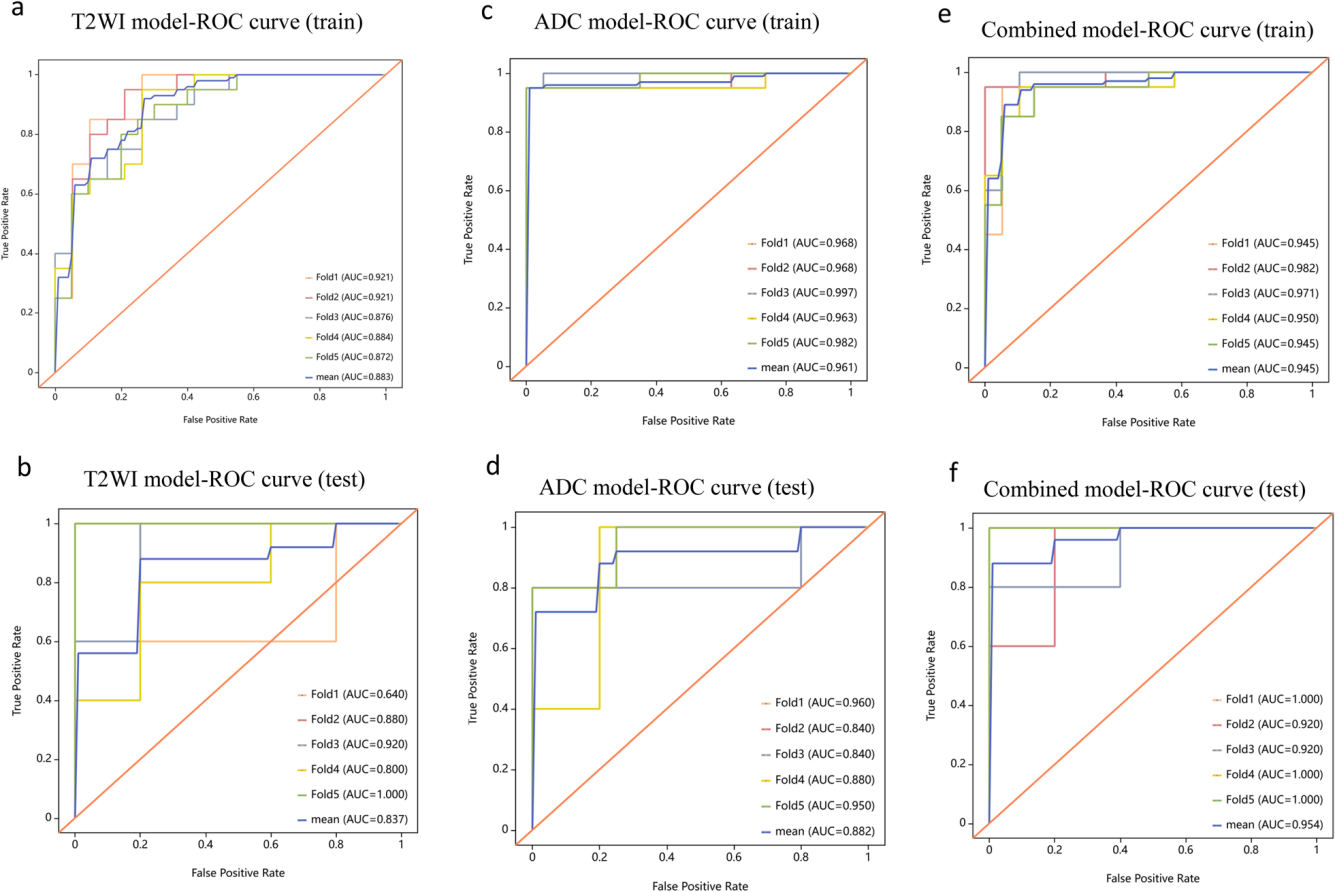
ROC curves of each fold and the best mean values for the T2WI model (a, b), ADC model (c, d) and combined model (e, f) on the training and test datasets

The radiomics analysis yielded good performance metrics in distinguishing between the BWS^UPD+IC2^ and BWS^IC1^ subtypes. The performance of all 104 algorithms for each model is shown in supplementary Table 1. Among them, the logistic regression applied to the T2WI model achieved the highest mean AUC of 0.837 (95% CI 0.618–1, accuracy 0.718, sensitivity 0.76, specificity 0.68) on the test dataset, demonstrating good discrimination capability. The random forest applied to the ADC model also showed comparable performance with the highest mean AUC of 0.882 (95% CI 0.664–1, accuracy 0.778, sensitivity 0.84, specificity 0.71) on the test dataset. The combined model, utilizing the quadratic discriminant analysis (QDA), demonstrated the highest diagnostic efficacy (AUC = 0.954, 95% CI 0.894–1, accuracy 0.9, sensitivity 0.92, specificity 0.88). ROC curves of each fold and the best mean values for the T2WI, ADC and combined models on the training and test datasets are presented on Fig. 8*.* For each fold, Delong tests of the best-performing algorithms in the three models showed no statistically significant differences in performance between the models (*P* > 0.05) (Table 5).

**Table 5:** Delong test results for the performance comparisons among the three best models for each fold

Model comparison	Fold 1	Fold 2	Fold 3	Fold 4	Fold 5
T2WI model vs. ADC model, *P* values	0.826	0.798	0.972	0.921	0.527
T2WI model vs. Combined model, *P* values	0.859	0.609	0.831	0.943	0.67
ADC model vs. Combined model, *P* values	1	0.46	0.904	0.96	0.91

## Discussion

### Overview of study findings

This study involved a cohort of BWS patients with three main molecular types and their renal MRI exams. We presented the first study of an MRI-based radiomics model to identify the genotype–phenotype correlations, including correlations of specific molecular aetiologies with nonmalignant renal abnormalities. Our results highlight the differences between the renal predisposition (BWS^UPD+IC1^) and non-predisposition (BWS^IC2^) molecular subtypes. Clear differences in clinical presentations, the renal volume, and incidence of nonmalignant renal abnormalities were identified between the two subtypes. Furthermore, renal radiomics features had a high performance to differentiate the two molecular types based on MRI images, with the highest AUC of 0.954 on the combined model using the T2WI and ADC features. Therefore, MRI radiomics in the kidney for BWS patients could serve as a good indicator for differentiating BWS epigenetic subtypes.

### Clinical manifestations on the renal predisposition and non-predisposition molecular subtypes

Previous studies have demonstrated a correlation between phenotype and genotype, revealing distinct clinical manifestations across different BWS subtypes. The IC1 hypermethylation subtype is characterized by a higher frequency of body overgrowth and visceromegaly, likely due to IGF2 overexpression [18], and a relatively lower frequency of ear creases, ear pits, facial nevus flammeus, and omphalocele [19]. The UPD subtype is primarily associated with hemihypertrophy, possibly due to asymmetrical post-mitotic mosaicism in patients with UPD or other factors affecting gene expression in 11p15.5 in a tissue-specific manner [20]. The IC2 hypomethylation subtype shows a high frequency of abnormal ear or facial features and a lower frequency of internal organ enlargement (particularly kidney and spleen) [11–13].

Our findings are partially consistent with previous studies, showing a lower frequency of omphalocele/umbilical hernia and ear creases/pits in the BWS^UPD+IC1^ subtype. However, no significant differences were observed between the subtypes in terms of hemihypertrophy, macrosomia, or facial nevus flammeus, which may be attributed to the small sample size and selection bias of patients with macroglossia.

### Renal and non-renal abnormalities on MRI

Children with BWS and Wilms tumor consistently present with bilateral nephromegaly, with the tumor always arising in the larger kidney [13, 21]. This highlights the importance of monitoring renal volume in BWS patients. Previous studies have used various imaging modalities, including ultrasound, CT, and MRI, to evaluate renal size and construct normative renal growth curves [17, 22, 23]. However, these studies estimated renal volume using a geometric model based on length, width, and depth measurements. In contrast, our study utilized 3D MRI-derived renal volumes based on VOI delineations, providing more precise measurements of renal volume in children.

Several studies have also assessed the relationship between renal volume and anthropometric parameters such as age, sex, height, weight, body surface area, and body mass index, demonstrating a strong correlation between renal volume and age (r = 0.85–0.91) [24–26]. A study found that nephromegaly was most common in the IC1 subtype with nearly all renal size measurements exceeding the + 2SD threshold, however, renal size varied widely in the UPD subtype and stayed near normal in the IC2 subtype [13]. Our findings partially align with this, showing larger renal volume and a weaker correlation between renal volume and age (r = 0.38) in the BWS^UPD+IC1^ subtype compared to BWS^IC2^ (r = 0.71), with IC1 showing the largest mean renal volume. However, unlike previous findings, the majority (91.84%) of our BWS patients across all three subtypes had mean total renal volumes exceeding the normal upper limit.

Renal morphological and structural anomalies are well-recognized features of BWS and included in diagnostic criteria. Nephrourological anomalies have been less frequently described, with reported incidences ranging from 28 to 61% [13]. These abnormalities are typically asymptomatic [9], with nephromegaly, collecting system abnormalities, renal cysts, and nephrolithiasis being the most common findings in BWS [12, 13]. In our study, the overall incidence of benign renal lesions was 22.4% (11/49), slightly lower than reported in the literature. Nephromegaly, hydronephrosis, and renal cysts were the most common, and the BWS^UPD+IC1^ subtype had a higher frequency of nonmalignant renal abnormalities compared to the BWS^IC2^ subtype. Notably, only 4 patients had nephromegaly identified through subjective assessment. In contrast, objective 3D MRI-drived measurements showed 45 patients had a total renal volume exceeding the normal upper limit for children, underscoring the value of VOI measurements in accurately assessing renal size. Additionally, nephrolithiasis may also be associated with BWS; however, MRI is limited in detecting microcalcifications. No Wilms tumor was found in our cohort, possibly due to the younger median age of patients undergoing macroglossia resection (median 15 months, range 6–48 months).

The difference in the risk of nonmalignant renal abnormalities between BWS^UPD+IC1^ and BWS^IC2^ subtypes is primarily due to the mutations of IGF2 and p57^KIP2^ (CDKN1C) which control growth and morphogenesis during kidney development [27, 28]. IGF2 overexpression in the IC1 subtype demonstrated striking nephromegaly [29], and the downregulation of p57^KIP2^ in the IC2 subytpe (loss of KvDMR1 methylation which might downregulate p57^KIP2^ expression) produced cystic renal disease [12]. The UPD subtype resulted in an increase in IGF2 expression and a decrease in p57^KIP2^ expression. However, in the IC2 subtype, p57^KIP2^ downregulation occurred without accompanying IGF2 overexpression, leading to milder renal manifestations potentially due to the compensation from reduced IGF2 levels. As demonstrated by Caspary et al., these two imprinted genes interacted antagonistically in a mouse model [28]. Compared to the double-mutant mice model (mimicking the UPD subtype), the disorganized development of tissues was exacerbated in an IGF2-dependent manner when p57^KIP2^ was absent as seen in the IC1 subtype. However, when IGF2 signaling was reduced, as in the IC2 subtype, the detrimental effects of p57^KIP2^ loss were mitigated.

Hepatic lesions in BWS include malignant hepatoblastoma and benign entities such as hepatic hemangiomas, focal nodular hyperplasia, mesenchymal hamartomas, and mesothelial inclusion cysts [30–32]. Among them, hepatic hemangiomas are rare in BWS, with only six reported cases confirmed by MS-MLPA testing in previous studies [31, 33–37]. Of these reports, the majority were UPD (four cases) subtype, followed by IC2 (two cases) subtype, without IC1 subytpe. However, in our study, the frequency of suspected hepatic vascular abnormalities was higher with five cases, predominantly in the UPD subtype (80%). This high frequency may potentially be linked to an increase in IGF2 expression in the UPD subtype, which could result in the change of cellular maturation within the developing liver [36, 38, 39]. Interestingly, our two cases may represent the first reports in BWS of (1) hepatic vascular anomalies in an IC1 patient and (2) concurrent renal—liver vascular anomalies in a UPD patient. A mesenteric cyst was identified in one BWS^UPD^ patient, consistent with isolated findings in BWS [40–42].

### Renal MRI radiomics

MRI demonstrates exceptional sensitivity to changes in biological tissue microstructure. Radiomic analysis comprehensively quantifies imaging data, extracting both first-order and higher-order features that reveal patterns not discernible to the naked eye [43]. In our study, the T2WI and ADC models demonstrated comparable predictive performance, with AUCs of 0.837 and 0.882, respectively. The combined model, however, achieved a higher AUC of 0.954, although there were no significant differences in performance among the three models.

These findings indicate that T2WI features effectively capture textural and intensity distribution properties relevant to distinguishing BWS genotypes with increased susceptibility to renal pathology. The two first-order features in the T2WI model reflect voxel intensity distributions, providing insights into tissue homogeneity and symmetry that correlate with structural changes in renal tissue. Furthermore, the inclusion of higher-order texture features derived from GLDM and GLSZM matrices enhances the model’s predictive capability by detailing complex spatial relationships and tissue heterogeneity.

The ADC model, consisting exclusively of texture features derived from GLDM, GLCM, and NGTDM matrices, highlights the role of DWI in assessing renal microstructure. GLDM and GLCM features capture variations in voxel intensity relationships, while NGTDM quantifies gray-level differences across neighborhoods [44], offering a nuanced assessment of microstructural changes in the renal parenchyma. Previous studies have demonstrated that DWI, quantified through ADC, is effective in detecting microstructural changes indicative of renal impairment [16].

Although the ADC model performed slightly better than the T2WI model, the voting count for T2WI features was higher in the combined model. We hypothesize that this may be attributed to the inclusion of critical first-order features, such as kidney volume, within the T2WI features.

### Limitations and future research directions

There were several limitations in this study. The first limitation is the small sample size. Future research should focus on larger cohorts with prospectively collected data to validate and improve the predictive accuracy of the radiomics models. Secondly, as this study was conducted at a single institution without external validation due to the rarity of BWS, there may be biases associated with patient selection and imaging protocols, limiting the ability to evaluate the generalization of our radiomics model. Further multi-center studies could address these issues. Thirdly, patients lacking the three main molecular types identified in other epigenetic tests—such as CDKN1C mutation (via WES, whole exome sequencing), chromosomal deletion and duplication (via CMA, chromosomal microarray analysis), and loss of heterozygosity (LOH)—were not included, which may reduce the clinical applicability of radiomics models. Expanding the inclusion criteria to incorporate these additional molecular subtypes would enable a more comprehensive assessment of the clinical utility of radiomics models across diverse genetic profiles.

In conclusion, the renal radiomics model derived from T2WI and ADC maps on MRI has potential as an alternative imaging tool for identifying renal predisposition genotypes of BWS^UPD+IC1^. Moreover, a larger renal volume was associated with these genotypes, which is linked to an increased risk of Wilms tumor. Additionally, certain clinical manifestations were more prevalent in BWS^IC2^, while non-malignant renal and non-renal abdominal abnormalities were more frequently observed in BWS^UPD+IC1^.

## Supplementary Information

Supplementary Table.

## Data Availability

The data reported in this article will be made available for noncommercial, academic purposes upon request.

## Abbreviations

ADC: Apparent diffusion coefficient
AUC: Area under the receiver operating characteristic curve
BWS: Beckwith–Wiedemann syndrome
BWS^IC2^: Molecular subtype IC2 with a low risk of renal involvement in BWS
BWS^UPD+IC1^: Molecular subtypes UPD and IC1 with a predisposition to renal involvement in BWS
CI: Confidence interval
DWI: Diffusion-weighted imaging
GLCM: Gray level of co-occurrence matrix
GLDM: Gray level of dependence matrix
GLRLM: Gray level of run length matrix
GLSZM: Gray level of size zone matrix
IC: Imprinting control
LASSO: The least absolute shrinkage and selection operator
MLPA: Multiplex ligation-dependent probe amplification
NGTDM: Neighbouring gray tone difference matrix
QDA: Quadratic discriminant analysis
ROC: Receiver operating characteristic
ROI: Region of interest
SD: Standard deviation
T2WI: T2-weighted imaging
UPD: Uniparental isodisomy
VOI: Voxel of interest
